# Recent Progress in Rice–*Xanthomonas oryzae* Interactions

**DOI:** 10.3390/biology14050471

**Published:** 2025-04-25

**Authors:** Yuting Qi, Qiong Rao, Chenglong Lu, Junyi Gong, Yuxuan Hou

**Affiliations:** 1 Zhejiang Key Laboratory of Biology and Ecological Regulation of Crop Pathogens and Insects, College of Advanced Agricultural Sciences, Zhejiang A&F University, Hangzhou 311300, China; 13140590443@163.com (Y.Q.); qiong.rao@zafu.edu.cn (Q.R.); 2 State Key Laboratory of Rice Biology and Breeding, China National Rice Research Institute, Hangzhou 311400, China; lclzbzkerban@163.com

**Keywords:** rice, *Xanthomonas oryzae*, effector, resistance gene, multi-omics

## Abstract

Rice bacterial blight (BB) and bacterial leaf streak (BLS), caused by *Xanthomonas oryzae* pv. *oryzae* (*Xoo*) and *Xanthomonas oryzae* pv. *oryzicola* (*Xoc*), respectively, are among the most devastating bacterial diseases threatening global rice production. The interactions between rice and *Xanthomonas oryzae* are complex and dynamic, involving recognition, attack, defense, and adaptation mechanisms enacted by both the rice host and the pathogens. This review summarizes recent advances in understanding rice–*Xanthomonas oryzae* interactions, focusing on infection models, pathogenic mechanisms, and immune responses elicited by *Xanthomonas oryzae*. Special attention is devoted to the roles of transcription activator-like effectors (TALEs) and non-TALE effectors in pathogenicity, the functions of resistance (*R*) genes in defense, and the interconnected molecular networks of interactions derived from multi-omics approaches. Understanding these interactions is essential for developing effective disease-resistance strategies and creating elite disease-resistant rice varieties.

## Simple Summary

Rice serves as a staple food crop for billions of people, but bacterial diseases like bacterial blight and bacterial leaf streak, caused by *Xanthomonas oryzae*, can severely reduce rice yields and threaten food security. This review explores how *Xanthomonas oryzae* infects rice plants and how rice defends against *Xanthomonas oryzae*, focusing on the roles of bacterial type III secretion effectors and host resistance genes, as well as the holistic insights into interaction mechanisms between the rice host and *Xanthomonas oryzae*. Modern genetic technologies, such as gene editing and marker-assisted selection, are discussed for being employed to develop next-generation disease-resistant rice varieties. These advances are crucial for reducing rice losses and ensuring stable food production.

## 1. Introduction

*Xanthomonas* is a genus of Gram-negative bacteria that infects approximately 400 host species, including rice, citrus, tomato, and pepper [1]. *Xanthomonas oryzae* pv. *oryzae* (*Xoo*) and *Xanthomonas oryzae* pv. *oryzicola* (*Xoc*), two closely related pathovars, cause bacterial blight (BB) and bacterial leaf streak (BLS) in rice, respectively [2]. BB is one of the most destructive rice diseases, leading to yield losses of 10–30%, with some cases exceeding 50% [3,4]. Similarly, BLS results in yield reductions of 8–32% [5]. The interactions between rice and *Xoo/Xoc* are intricate and dynamic, with the pathogens attempting to bypass the host’s defense mechanisms while the rice plant employs immune responses to resist infection. This review provides a comprehensive overview of rice–*Xoo/Xoc* interactions and summarizes recent advancements regarding the immune responses induced by type III secreted effectors and the application of multi-omics technologies to elucidate the molecular mechanisms of these interactions.

## 2. *Xanthomonas oryzae* Infection Models

**Figure 1:**
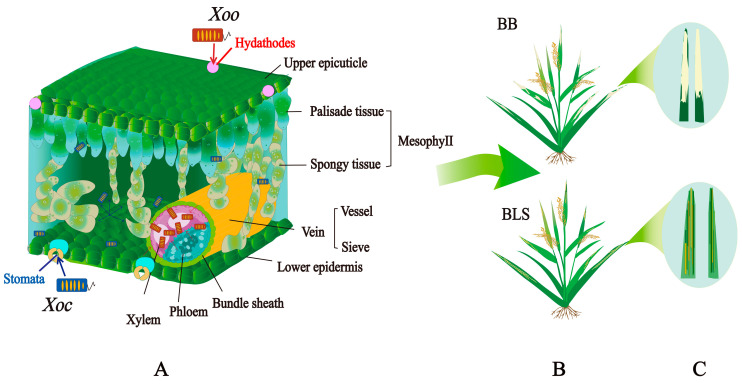
Infection modes of *Xoo* and *Xoc* in rice. (**A**) Schematic representation of the infection modes of *Xoo* and *Xoc* in rice leaf tissue. (**B**) Symptoms of BB and BLS caused by *Xoo* and *Xoc*, respectively. (**C**) The magnified images of BB and BLS symptoms.

*Xoo* typically enters rice leaves through hydathodes at the edges and tips or through wounds. The bacteria multiply in the intercellular spaces of parenchyma cells and spread to the xylem, forming a beaded liquid on the leaf surface after a few days [6]. *Xoo* interacts with xylem parenchyma cells, moving vertically through the leaf via primary veins and laterally through commissural veins (Figure 1A). In contrast, *Xoc* penetrates the leaf mainly through stomata, multiplies in the sub-stomatal cavity, and remains confined to the apoplast of the mesophyll tissue without invading the xylem (Figure 1A) [2]. It exudes yellow beads or strands from natural openings, contributing to disease spread [6,7].

Due to their different infection methods, BB and BLS are easily distinguishable in the early stages but may appear similar later. BB symptoms begin as small, green, water-soaked spots at the leaf edges that turn into gray lesions (Figure 1B,C). BLS starts with water-soaked lesions between veins that form into translucent yellow streaks (Figure 1B,C). As both diseases progress, their symptoms can overlap, leading to confusion. Both pathogens often coexist in rice fields, with individual leaves displaying symptoms of both diseases [6,8].

## 3. Type III Secreted Effectors of *Xanthomonas*—TALEs

**Figure 2:**
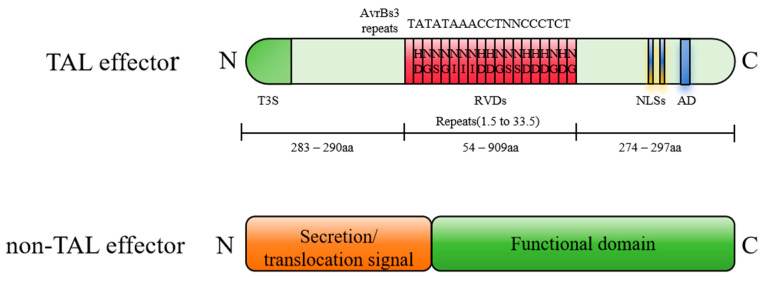
Structural features of TALEs and non-TALEs.

During infection, *Xanthomonas* secretes effectors into host cells primarily via the type III secretion system (T3SS), which is crucial for pathogenesis [9,10]. Most secreted effectors, known as type III secreted effectors (T3SEs), include transcription activator-like effectors (TALEs). TALEs are notable for inducing the expression of host target genes in the nucleus [11]. TALE proteins exhibit specific structural features: (1) The NH2-terminal region is highly conserved and includes a T3SS signal (T3S) for translocating TALEs to the host cytoplasm; (2) The COOH-terminal region contains nuclear localization signals (NLSs) that transport TALEs to the nucleus and a conserved acidic activation domain (AD) for gene transcription activation (Figure 2). Moreover, TALEs have tandem repeat regions (RR) and repeat variable di-residues (RVDs) at positions 12 and 13 that interact specifically with host DNA, binding to effector-binding elements (EBEs) in gene promoters to activate their expression (Figure 2).

The first TALE to be characterized was AvrBs3 from *Xanthomonas campestris* pv. *Vesicatoria,* which triggers Bs3-mediated resistance in peppers [12]. AvrBs3 homologs were subsequently found in *Xoo*, *Xoc*, *Xanthomonas campestris* pv. *campestris,* and other *Xanthomonas* species [13,14,15,16]. Some *Xanthomonas* genomes contain fewer than 6 TALEs, while others, like *Xoo* and *Xoc*, can have over 10, with a maximum of 28 [17]. It has been confirmed that PthXo1, PthXo2, PthXo3, and AvrXa7 are significant TALEs of *Xoo*, accounting for more than 80% of the virulence for rice, as quantified by lesion length, when compared to the full virulence associated with wild-type strains [10,18]. Truncated TALE genes, previously thought to be pseudogenes, have been identified in *Xoo/Xoc* strains and confirmed as truncated TALEs or interfering TALEs (iTALEs). Unlike typical TALEs, iTALEs have 45 or 129 bp deletions in the N-terminal region and lack C-terminal AD domains [19].

## 4. Type III Secreted Effectors of *Xanthomonas*—Non-TALEs

In addition to TALEs, T3SS includes non-TALE effectors. Non-TALEs are found in most *Xanthomonas* species and are primarily composed of a secretion translocation signal and a functional domain (Figure 2). Eighteen non-TALEs are universally present in *Xanthomonas*. Genome sequence analysis of *Xoo* and *Xoc* strains revealed that non-TALEs are highly conserved, although their numbers vary. Specifically, *Xoo* strains KACC10331, MAFF311018, and PXO99^A^ contain 19, 24, and 20 non-TALEs, respectively, while *Xoc* strain BLS256 has 26 [20,21,22].

Some effectors are unique to *Xanthomonas oryzae*, including XopU, XopW, XopY, and XopAB. Notably, XopT and XopAF are exclusively present in *Xoo* and *Xoc*, respectively, while XopO and XopAJ are unique to *Xoc* and *Xanthomonas citri* subsp. *viticola* [18,23,24]. AvrBs2, the first described non-TALE in *Xanthomonas campestris* pv. *vesicatoria*, is highly conserved [25,26]. Furthermore, XopN has shown similar pathogenicity to AvrBs2 in the GX01 strain of *Xoc*. In the PX099^A^ strain, a triple mutant (XopZ, XopN, XopV) exhibited shorter lesion lengths, but virulence was restored by reintroducing these effectors in the Kitaake variety [27].

## 5. TALEs-Induced Rice Immunity to *Xanthomonas oryzae*

The plant immune system serves as a barrier against pathogen infection and comprises pathogen-associated molecular pattern (PAMP)-triggered immunity (PTI) and effector-triggered immunity (ETI) [28]. In PTI, plant cells employ pattern recognition receptors (PRRs) to recognize PAMPs and initiate basal immune responses. For example, FLAGELLIN SENSITIVE2 (OsFLS2) perceives bacterial flagellins, activating downstream defense signaling pathways [29]. In contrast, ETI involves resistance (R) proteins such as nucleotide-binding leucine-rich repeat (NLR)-type protein Xa1, which specifically recognizes TALEs and triggers stronger immune responses [30]. The presence of functional R proteins leads to ETI, whereas their absence results in effector-triggered susceptibility (ETS) [31]. The interplay between pathogen effectors and their corresponding R proteins reflects a molecular confrontation between the pathogen and the host plant. In *Xoo/Xoc*–rice interactions, TALEs with targeted *R* genes are key components determining rice resistance or susceptibility to *Xoo/Xoc*.

For ETI, rice *R* genes targeted by TALEs of* Xoo* have been identified and cloned. *Xa1 *is the first cloned NLR-type *R* gene. *Xa1* can be recognized by multiple TALEs, such as PthXo1, Tal4, and Tal9d. It was reported that *Xa1*-mediated resistance triggered by TALEs can be suppressed by iTALEs [30,32]. *Xa2/Xa31*, *Xa14*, and *Xa45 *have also been successfully identified and cloned as alleles of *Xa1, *exhibiting similar functional properties to* Xa1 *(Table 1) [19,32,33]. Additionally, executor (*E*) resistance genes, including *Xa7*, *Xa10*, *Xa23*, and *Xa27*, are induced by altering the promoter structure, allowing recognition by their corresponding TALEs AvrXa7/PthXo3, AvrXa10, AvrXa23, and AvrXa27, thereby conferring resistance against *Xoo* (Table 1) [34,35,36,37]. Moreover, some TALEs, such as Tal7, Tal9A, and Tal1C, have been identified, but their molecular mechanisms of interacting proteins in rice remain unclear [38,39].

The genetics of resistance to *Xoc* are complex, and available resources on resistance are limited, resulting in significantly slower research progress compared to *Xoo*. *Rxo1*, the first cloned non-host resistance gene in maize, encodes a NLR protein and confers resistance to *Xoc* when introduced into rice [57,58]. *Xo1*, an allele of *Xa1*, recognizes diverse TALEs from both *Xoo* and *Xoc *(Table 1) [59]. However, the resistance mediated by *Xo1* can be suppressed by interfering TALEs (iTALEs). Additionally, *Xo1* only confers resistance to African *Xoc* strains and is ineffective against Asian *Xoc *strains [32,59,60]. Notably, the “truncTALE” Tal2h effector from *Xoc *strain BLS256 can suppress *Xo1*-mediated resistance [59,61].

For ETS, rice susceptibility (*S*) genes are genetically dominant, and their expression is induced upon pathogen infection. The induction of SWEET (Sugar Will Eventually Be Exported Transporter) genes facilitates pathogen nutrient acquisition and promotes disease development. For example, the TALE PthXo1 directly binds to EBE in the promoter of *OsSWEET11* (*Xa13*/*Os8N3*), inducing its expression and conferring susceptibility to *Xoo *[39,43,49,62,63]. Similarly, the *OsSWEET14* (*Xa41*/*Os11N3*) promoter is targeted by multiple TALEs, including AvrXa7, PthXo3, TalC, and Tal5, leading to its activation [50,51,52,64]. Additionally, the susceptibility gene *SWEET13* (*Xa25/Os12N3*) can be activated by PthXo2 and PthXo2-like effectors, which bind to variable EBEs in its promoter (Table 1) [44,65]. In contrast, mutations in the EBEs of their recessive alleles, such as* xa13*, *xa41*, and *xa25*, prevent recognition by the aforementioned TALEs, disrupting pathogen colonization and conferring resistance to *Xoo* in rice (Table 1) [44,45,66]. *OsSWEET12* and *OsSWEET15* have also been identified as *S *genes during *Xoo* infection, as their expression can be induced by the artificial TAL effectors ArtTAL12 and ArtTAL15 [52].

Many non-SWEET *S* genes play critical roles during *Xoo/Xoc* infection. The gamma subunit of the basal transcription factor, TFIIAγ5 (also known as Xa5), binds directly to the TFB region of TALEs, forming a complex that facilitates the transcription of TALE-activated genes (Table 1) [54]. However, the mutant variant *xa5*, which encodes a naturally occurring V39E variant of TFIIAγ5, cannot interact with TALEs, reducing the expression of TALE-driven *S* or *E* genes to enhance rice resistance (Table 1) [67,68]. In the absence of *TFIIAγ5*, another *TFIIAγ* gene, *OsTFIIAγ1*, can be activated by PthXo7, explaining the reason that PthXo7-containing *Xoo* strains overcome *xa5*-mediated resistance (Table 1) [67]. Interestingly, *qBlsr5a* was identified as an allele of *xa5*, which confers resistance to *Xoc* [69]. Additionally, *OsTFX1*, encoding a basic leucine zipper (bZIP) transcription factor, is induced by PthXo6 and TalBMAI1 (Table 1) [46,55]. TalBMAI1 also activates *OsERF#123*, an AP2/ERF transcription factor gene that contributes to susceptibility to African *Xoo* strains (Table 1) [55]. In rice–*Xoc* interactions, the sulfate transporter gene O*sSULTR3;6* is targeted by Tal2g and serves as a major *S* gene for *Xoc* (Table 1) [56].

## 6. Non-TALE-Induced Rice Immunity to *Xanthomonas oryzae*

The targets and molecular mechanisms for most non-TALEs in plant cells remain largely unknown, and a few non-TALEs in *Xoo *have been characterized (Table 2). It was reported that the interaction between non-TALE XopN and OsVOZ2 promotes rice susceptible to *Xoo*, while the interaction between XopN and OsXNP is speculated to induce calcium deposition and hydrogen peroxide accumulation against *Xoo* (Table 2) [27,70,71]. Additionally, XopR of *Xoo* interacts with OsBIK1, suppressing PAMP-triggered stomatal closure in *Arabidopsis* (Table 2) [72]. Other non-TALEs, such as XopY (Xoo1488), XopAA (Xop2875), and XopK, interact with OsRLCK185, OsBAK1, and OsSERK1, respectively. OsRLCK185 is involved in chitin-induced immune responses. Xoo1488 suppresses chitin-induced MAPK activation by inhibiting the phosphorylation of OsRLCK185 [73]. OsBAK1 is a key component of both microbe-associated molecular patterns (MAMPs) and brassinosteroid (BR) receptors, suggesting that the virulence activity of Xoo2875 is likely mediated by the inhibition of OsBAK1 [74]. XopK directly ubiquitinates the receptor kinase OsSERK2, leading to its degradation and thereby suppressing the immune response triggered by PAMP [75]. XopP interacts with the rice E3 ubiquitin ligase OsPUB44 to inhibit rice resistance to *Xoo* (Table 2) [76]. Furthermore, XopL interacts with ferredoxin proteins (NbFd) in non-host plants, promoting reactive oxygen species (ROS) burst and inducing cell death (Table 2) [77]. XopZ was found to interact with ORP1C in *Xoo* strain PXO99^A^, but ROS burst and PTI marker gene expression data suggest that ORP1C is not involved in the PTI pathway in rice (Table 2) [78]. These findings highlight the potential for cooperation among multiple non-TALEs and their diverse physiological functions in the host, particularly in modulating innate immune responses.

## 7. Whole Picture of Rice–*Xanthomonas oryzae* Interaction Mechanisms from Multi-Omics View

Although many bacterial virulence factors and rice resistance genes have been identified or cloned in rice–*Xanthomonas oryzae* interactions as described above, the molecular mechanisms behind these interactions remain fragmented, with most studies focusing on individual components rather than systemic networks. Over the past two decades, genome-derived multi-omics studies have gradually evolved and expanded. Numerous plant functional genomics studies, which integrate the generation of transgenic and mutant plants with parallel analyses of mRNA expression, protein levels, and metabolic profiles, have been applied to uncover the complex molecular basis underlying rice immunity against *Xanthomonas oryzae* [79,80,81,82]. The systems-level understandings derived from integrated multi-omics reveal interconnected molecular networks and lay the groundwork for the breeding of *Xoo*/*Xoc*-resistant rice varieties, as well as broad-spectrum disease-resistant cultivars. Furthermore, they offer valuable data to develop novel organic pesticides.

Genome re-sequencing of diverse rice varieties can be conducted to comprehensively reveal genomic variations and interactions, facilitating the discovery of novel genes associated with disease resistance . Genome-wide association study (GWAS) analysis can validate the known resistance genes and identify novel sites to expand the current resistance gene pool. A total of 77 and 7 loci associated with *Xoo* and *Xoc* resistance, respectively, were identified with the GWAS analysis of 895 accessions from the 3000 Rice Genomes Project (3K RGP) (Table 3). Among the loci, seven for *Xoo* resistance were co-localized with known *Xoo* resistance genes, and one locus for *Xoc* resistance overlapped with a previously reported *Xoc* resistance QTL. The remaining novel loci encompass several defense-related genes potentially involved in* Xoo* and* Xoc* resistance [83]. Through another GWAS involving 340 accessions from the 3K RGP, a total of 11 QTLs associated with *Xoo* resistance were identified (Table 3). Eight of these resistance loci were mapped to relatively small genomic intervals, consistent with previously reported QTLs or resistance genes. Linear regression analysis revealed a significant correlation between bacterial blight lesion length and the number of favorable resistance alleles [84]. Furthermore, whole genome sequences can provide insights into phylogenetic relationships and help predict genes associated with strain-specific virulence factors and behaviors. A GWAS of 172 global indica rice germplasm infected by representative strains from six *Xoo* races (China and the Philippines) highlighted the importance of chromosomes 11 and 12 in the evolution of rice disease resistance (Table 3). The hotspot region on chromosome 11 contained 89.6% of significant SNPs associated with resistance to race P1, while the chromosome 12 hotspot encompassed 85.3% of SNPs linked to race P9a resistance [85].

Proteomic analyses of resistant and susceptible rice cultivars during pathogen infection have revealed key proteins associated with defense mechanisms [93]. Time-course proteomic profiling of susceptible rice (RLX) leaves at 3, 6, and 12 h post-inoculation identified critical virulence-related proteins in *Xoo*, including carbohydrate metabolism enzymes (hexose phosphate mutase, inositol monophosphatase), arginase, and septum site-determining protein (Table 3) [86]. Comparative proteomics between wild-type *Xoc* and its *rpfF* mutant (encoding diffusible signal factor synthase) demonstrated DSF’s regulatory role in virulence through nitrogen transfer, protein folding, ROS scavenging, and flagellum formation (Table 3) [87]. In another study comparing incompatible (H471-PXO99A) and compatible (HHZ-PXO99A) interactions, 374 host and 117 pathogen differentially abundant proteins (DAPs) were identified, predominantly involved in secondary metabolism and virulence, respectively. Further, it was demonstrated that phytoalexin and salicylic acid (SA) signaling pathways were activated faster in the incompatible interaction than in the compatible interaction (Table 3) [88].

Transcriptomic profiling serves as a powerful tool for the systematic identification of defense response (DR) genes involved in rice–*Xoo* interactions. RNA-sequencing analysis of susceptible rice inoculated with two *Xoc* strains (hypervirulent HGA4 and hypovirulent RS105) revealed distinct temporal patterns of differentially expressed genes (DEGs) at 12 hours (PTI phase) and at 3 days post-inoculation (ETI/ETS phase) (Table 3) [89]. The early PTI stage was characterized by conserved DEGs mediating broad-spectrum basal defense, while the late stage showed the predominant regulation of TALE and DR genes. Parallel investigations in *Xoo*–rice interactions demonstrated that mutants of host-induced virulence factors (*ΔxanA* and *Δimp*) similarly disrupted photosynthetic efficiency, redox homeostasis, and secondary metabolite biosynthesis pathways (Table 3) [86]. Furthermore, temperature-dependent transcriptomic analysis demonstrated that WRKY and ERF transcription factor families mediate a temperature-sensitive defense-growth trade-off in rice. Under low-temperature condition, plants sustained the robust activation of defense pathways against* Xoo* infection. Conversely, elevating temperature induced a physiological shift where resources were preferentially allocated to growth and reproductive processes, resulting in attenuated pathogen responses (Table 3) [90].

When plants are infected by pathogens, they synthesize specialized metabolites. These metabolites generally fall into three categories: primary metabolites, secondary metabolites, and plant hormones [94]. Among these, secondary metabolites such as terpenoids, phenolics, nitrogen-containing compounds, sulfur-containing compounds, and others play a critical role in plant interactions with biotic and abiotic environments and act as modulators of plant defense [95,96,97]. By analyzing and comparing the metabolic characteristics of three rice varieties—resistant (IRBB27), susceptible (IR24), and wild-type (CG154)—in response to bacterial leaf blight, various defense-related metabolites were identified, including amino acids, flavonoids, alkaloids, terpenes, nucleotide derivatives, organic acids, inorganic compounds, fatty acids, and lipid derivatives. Among these, key metabolites such as flavonoids, terpenes, and phenolic compounds showed significantly higher levels in resistant varieties [91]. Rice variety CBB23, which carries the *Xa23* resistance gene, was inoculated with *Xoo* strains AH28 and PXO99^A^. Metabolomics analysis showed that a large amount of alkaloid and terpenoid metabolite content decreased significantly after inoculation with AH28 compared to inoculation with PXO99^A^, while the content of amino acids and their derivatives significantly increased [92].

Generally, metabolomics provides a comprehensive analysis of all small molecules within an organism, positioned at the phenotypic endpoint of the omics cascade. It captures the results of an informative sequence starting from the genome and extending through the transcriptome and proteome, offering critical insights into the biochemical basis of plant–pathogen interactions.

## 8. Concluding Remarks and Future Perspectives

Significant progress has been made in understanding the interaction between rice and *Xoo*, particularly in identifying and cloning pathogenic effectors and the corresponding rice *R* genes. However, there exists a notable gap in knowledge concerning the effectors of *Xoc* and their targeted *R* genes. Therefore, it is imperative to explore new effectors and their interactions with rice for *Xoc* infection.

To date, a total of 44 *R* loci conferring resistance to BB have been identified, with 15 of these *R* genes having been successfully cloned [98]. Among the 15 *R* genes, *Xa4*, *xa5*, *Xa7*, *xa13*, *Xa21, *and *Xa23* have demonstrated strong and broad-spectrum resistance and been widely used in disease-resistant breeding. However, in recent years, due to the evolution of *Xoo*, many previously resistant rice varieties have lost their effectiveness, highlighting the urgent need to identify new resistance genes and develop new disease-resistant rice varieties adapted to the emerging *Xoo* strain [99].

**Figure 3:**
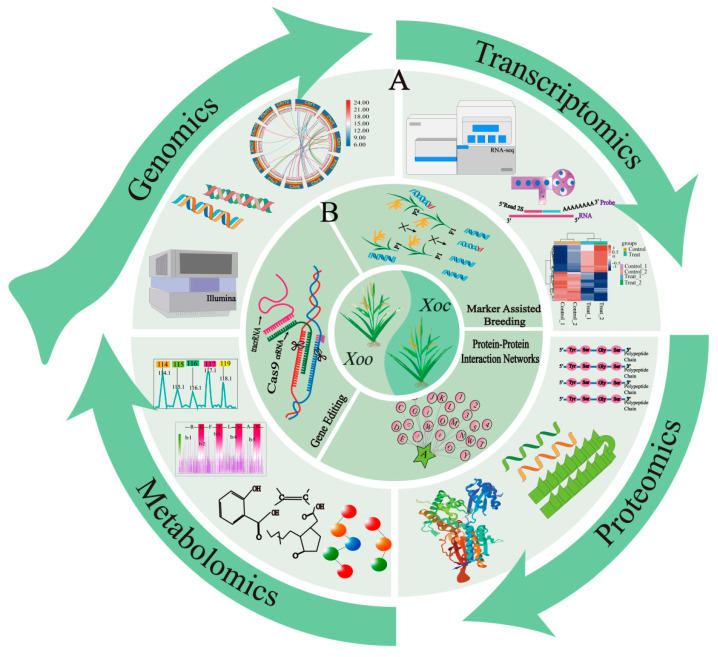
Multi-omics research and modern biotechnology strategies in rice–*Xoo/Xoc *interactions. (**A**) Application of four omics approaches (genomics, transcriptomics, proteomics, and metabolomics) in rice–*Xoo/Xoc* interactions. (**B**) Utilization of modern molecular biotechnology for resistance breeding against *Xoo/Xoc*.

To address this challenge, gene editing strategies and molecular marker-assisted selection (MAS) have been employed to create broad-spectrum disease-resistant rice varieties (Figure 3). The disruption of the binding elements for PthXo3, AvrXa7, and PthXo2 within the promoter regions of the *OsSWEET14* and *OsSWEET13* genes through TALEN technology has demonstrated significant resistance to BB [44]. The application of CRISPR/Cas9 technology to target and mutate EBEs of *OsSWEET11* and *OsSWEET14* in the rice cultivar Kitaake has successfully generated novel rice cultivars. These cultivars, exhibiting mutations in PthXo2-EBE, along with mutations in PthXo1-EBE and PthXo3-EBE, have been shown to confer a broad spectrum of resistance to *Xoo* infection [53]. Prime Editor (PE) technology addresses the limitations of low homology-directed repair (HDR) efficiency and significantly enhances gene editing precision. Gupta et al. successfully employed the PE5max system to introduce EBE from *OsSWEET14 *into the promoter of the dysfunctional *R* gene x*a23*, creating a functional *R* gene, *Xa23^SW14^.* This modification led to dominant resistance, effectively protecting rice against all *Xoo* strains carrying pthXo3/avrXa7. Additionally, they converted *TFIIAγ5* to *xa5*, which offers protection against all Asian* Xoo* strains except those carrying pthXo1 [100]. Further, the double-mutant lines obtained by converting *TFIIAγ5* to *xa5* and *xa23* to *Xa23* using the duplex PE system exhibited robust broad-spectrum resistance against multiple *Xoo* strains [101]. These studies demonstrate the potential of PE technology for precise genetic modifications to enhance disease resistance in crops.

Similarly, several modified disease resistance genes targeting *Xoc* have been identified in rice. EBEs of *OsSWEET11*, *OsSWEET14*, and *OsSULTR3;6 *in the rice cultivars Guihong 1 and Zhonghua 11 were precisely edited using CRISPR/Cas9 technology. This resulted in the development of the GT0105 (derived from Guihong 1) and ZT0918 (derived from Zhonghua 11) rice varieties, which exhibited significantly enhanced resistance to both *Xoo* and *Xoc* strains while maintaining agronomic traits comparable to their wild-type counterparts [102,103]. These findings demonstrate that precise editing of EBEs and *S* genes in the rice genome can effectively reduce disease incidence without compromising plant performance.

MAS breeding is increasingly used to enhance crop resistance (Figure 3). The success of MAS relies on the availability of strong genes and effective molecular markers. Scientists have developed markers like PR-Bs3, Xa27Fun, Xa23Fun, and MX7 based on *E* gene promoter characteristics, leading to the creation of rice varieties with improved resistance [104,105]. Resistance in *E* genes largely depends on the EBEs in their promoters. It was reported that adding six EBEs to the *Xa27* promoter allowed the susceptible rice cultivar Kitaake to gain broad-spectrum resistance to both *Xoo* and *Xoc* [106]. Similarly, using a promoter with five EBEs to drive *Xa10* expression also provideed broad-spectrum resistance to *Xoo* [107].

The elucidation of the molecular mechanisms underlying rice disease resistance, especially through the application of multi-omics approaches, remains insufficiently explored. The study of plant–pathogen protein interactions (PPI) is crucial for understanding plant diseases and developing effective control strategies (Figure 3). Researchers have constructed plant–pathogen interactomes through predictions and experimental methods. For instance, a study identified 3074 potential PPIs between *Ralstonia solanacearum* and *Arabidopsis thaliana*, highlighting the importance of pathogen-targeted proteins in the *Arabidopsis* PPI network [108]. A computational framework based on structural information has also been proposed to predict PPIs, which is more effective than sequence-based methods [109]. Experimental studies are revealing PPIs as well. Two pathogens and approximately 8000 *Arabidopsis* proteins were used to create an immune system protein interaction network, finding critical links between effectors and immune receptors [110]. Additionally, researchers developed a network of virulence effector protein interactions involving both *ascomycete* pathogens and *Arabidopsis* host proteins, identifying converging host proteins [110,111]. An ABA–T3SE interactome network was also established to study how T3SEs influence abscisic acid responses [112].

However, studies on pathogen–rice interaction networks, particularly those involving *Xanthomonas oryzae*, remain limited. Developing comprehensive interaction networks between pathogens and rice could help clarify the relationships between effector proteins and rice genes, paving the way for the identification of novel resistance (*R*) genes and a deeper understanding of the associated mechanisms.

In summary, the rice–*Xoo*/*Xoc* pathosystem is a powerful model for advancing disease control research. By combining genomics, proteomics, transcriptomics, and metabolomics, this system provides a multi-omics framework to dissect rice resistance genes and their regulatory networks (Figure 3). A thorough understanding of the interactions between rice and *Xanthomonas oryzae* is crucial for designing more effective and sustainable strategies to combat bacterial diseases in rice.

## Figures and Tables

**Table 1:** Rice genes targeted by TALEs.

Tale-Targeted (*R*/*S *Gene)		Encoding Products	Matched TALEs	References
Resistance	*Xa1* *Xo1* *Xa2/31* *Xa14* *Xa45*	NLR	Multiple TALEs, iTALEs/truncTALE	[19,30,33,40]
	* Xa7 *	Executor	AvrXa7, PthXo3	[37,41]
	* Xa10 *	Executor	AvrXa10	[35]
	* Xa23 *	Executor	AvrXa23	[42]
	* Xa27 *	executor	AvrXa27	[34]
	* xa13 *	Sweet transporter	PthXo1	[43]
	* xa25 *	Sweet transporter	PthXo2	[44]
	* xa41 *	Sweet transporter	AvrXa7, PthXo3, Tal5, TalC	[45]
	* xa5 *	TFIIA transcription factor	AvrXa5, PthXo7	[46,47,48]
Susceptibility	* OsSWEET11(Xa13/Os8N3) *	Sweet transporter	PthXo1	[49]
	* OsSWEET14(Xa41/Os11N3) *	Sweet transporter	AvrXa7, PthXo3, TalC, Tal5	[50,51,52]
	* OsSWEET13(Xa25/Os12N3) *	Sweet transporter	PthXo2	[44,53]
	* OsSWEET12 *	Sweet transporter	ArtTAL12	[52]
	* OsSWEET15 *	Sweet transporter	ArtTAL15	[52]
	* OsTFIIAγ5 *	Gamma subunit of rice basal transcription factor	Multiple TALEs	[54]
	* OsTFIIAγ1 *	Gamma subunit of rice basal transcription factor	PthXo7	[46]
	* OsTFX1 *	bZIP transcription factor	PthXo6 TalB_MAl1_	[46,55]
	* OsERF#123 *	AP2/ERF transcription factor	TalB_MAl1_	[55]
	* OsSULTR3;6 *	Sulfate transporter	Tal2g	[56]

**Table 2:** Rice genes interacted with non-TALEs.

Rice Genes (Interaction Genes)	Encoding Products	Matched TALEs	References
* OsVOZ2, OsXNP *	Vascular plant one zinc finger protein 2, putative thiamine synthase	XopN	[27,70,71]
* OsBIK1 *	Receptor-like kinases	XopR	[72]
* OsRLCK185 *	Receptor-like kinase	XopY	[73]
* OsBAK1 *	Receptor-like kinase	XopAA	[74]
* OsSERK2 *	Somatic embryogenic receptor kinase 2	XopK	[75]
* OsPUB44 *	Ubiquitin E3 ligase	XopP	[76]
* NbFd *	Ferredoxin protein	XopL	[77]
* OsORP1C *	Oxysterol-binding related protein	XopZ	[78]

**Table 3:** Rice genes targeted by TALEs.

Omics	Rice Varieties	* Xanthomonas oryzae *	Main Conclusion	References
Genomics	895 accessions from the 3K RGP	*Xoo* *Xoc*	7 and 77 loci linked to resistance for *Xoo* and *Xoc*, respectively, were identified	[83]
Genomics	340 accessions from the 3K RGP	* Xoo *	11 loci linked to resistance against *Xoo* were identified	[84]
Genomics	172 *indica* rice	* Xoo *	Chromosomes 11 and 12 were important for the evolution of rice resistance for *Xoo*	[85]
Proteomics	IR24	* Xoo *	Carbohydrate-metabolizing enzymes play a key roles in rice*–Xoo* interactions	[86]
Proteomics	Shanyou63	* Xoc *	DSF may play an important role in *Xoc* virulence and growth	[87]
Proteomics	H471 and HHZ	* Xoo *	Phytoalexin and SA signaling pathways were activated faster in the incompatible interaction than in the compatible interaction	[88]
Transcriptomics	ZH11	* Xoc *	Early PTI: conserved DEGs drive basal defense; Late ETI/ETS: TALE targets and specialized DR genes prevail	[89]
Transcriptomics	IR24	* Xoo *	The *ΔxanA* and *Δimp* mutants dysregulated photosynthesis, redox balance, and secondary metabolism	[86]
Transcriptomics	IR24	* Xoo *	Rice plants tend to shift their focus from defensive responses to growth and reproduction at high temperatures	[90]
Metabolomics	IRBB27, * Oryza minuta * * - * CG154 , IR24	* Xoo *	Key metabolites such as flavonoids, terpenes, and phenolic compounds showed significantly higher levels in resistant varieties	[91]
Metabolomics	CBB23	* Xoo *	Metabolites such as alkaloids and amino acid were involved in rice defense against *Xoo*	[92]

## Data Availability

No new data were created or analyzed in this study.

## References

[1] Ryan R.P., Vorhölter F.J., Potnis N., Jones J.B., Van Sluys M.A., Bogdanove A.J., Dow J.M. (2011). Pathogenomics of *Xanthomonas*: Understanding bacterium-plant interactions. Nat. Rev. Microbiol..

[2] Niño-Liu D.O., Ronald P.C., Bogdanove A.J. (2006). *Xanthomonas oryzae* pathovars: Model pathogens of a model crop. Mol. Plant Pathol..

[3] Joshi J.B., Arul L., Ramalingam J., Uthandi S. (2020). Advances in the *Xoo*-rice pathosystem interaction and its exploitation in disease management. J. Biosci..

[4] Hsu Y.C., Chiu C.H., Yap R., Tseng Y.C., Wu Y.P. (2020). Pyramiding Bacterial Blight Resistance Genes in Tainung82 for Broad-Spectrum Resistance Using Marker-Assisted Selection. Int. J. Mol. Sci..

[5] Liu W., Liu J., Triplett L., Leach J.E., Wang G.L. (2014). Novel insights into rice innate immunity against bacterial and fungal pathogens. Annu. Rev. Phytopathol..

[6] Mew T.W., Alvarez A.M., Leach J.E., Swings J. (1993). Focus on bacterial blight of rice. Plant Dis..

[7] Wang L., Makino S., Subedee A., Bogdanove A.J. (2007). Novel candidate virulence factors in rice pathogen *Xanthomonas oryzae* pv. oryzicola as revealed by mutational analysis. Appl. Env. Microbiol..

[8] Jiang N., Yan J., Liang Y., Shi Y., He Z., Wu Y., Zeng Q., Liu X., Peng J. (2020). Resistance Genes and their Interactions with Bacterial Blight/Leaf Streak Pathogens (*Xanthomonas oryzae*) in Rice (*Oryza sativa* L.)-an Updated Review. Rice.

[9] Timilsina S., Potnis N., Newberry E.A., Liyanapathiranage P., Iruegas-Bocardo F., White F.F., Goss E.M., Jones J.B. (2020). *Xanthomonas* diversity, virulence and plant-pathogen interactions. Nat. Rev. Microbiol..

[10] Xu X., Li Y., Xu Z., Yan J., Wang Y., Wang Y., Cheng G., Zou L., Chen G. (2022). TALE-induced immunity against the bacterial blight pathogen *Xanthomonas oryzae* pv. oryzae in rice. Phytopathol. Res..

[11] Boch J., Bonas U. (2010). *Xanthomonas* AvrBs3 family-type III effectors: Discovery and function. Annu. Rev. Phytopathol..

[12] Bonas U., Stall R.E., Staskawicz B. (1989). Genetic and structural characterization of the avirulence gene avrBs3 from *Xanthomonas campestris* pv. vesicatoria. Mol. Gen. Genet..

[13] Swarup S. (1991). Isolation of pathogenicity genes from Xanthomonas species and study of their regulation.

[14] Swarup S., Yang Y., Kingsley M.T., Gabriel D.W. (1992). An *Xanthomonas* citri pathogenicity gene, pthA, pleiotropically encodes gratuitous avirulence on nonhosts. Mol. Plant Microbe Interact..

[15] Hopkins C.M., White F.F., Choi S.H., Guo A., Leach J.E. (1992). Identification of a family of avirulence genes from *Xanthomonas oryzae* pv. oryzae. Mol. Plant Microbe Interact..

[16] Yang B., White F.F. (2004). Diverse members of the AvrBs3/PthA family of type III effectors are major virulence determinants in bacterial blight disease of rice. Mol. Plant Microbe Interact..

[17] Scholze H., Boch J. (2011). TAL effectors are remote controls for gene activation. Curr. Opin. Microbiol..

[18] White F.F., Potnis N., Jones J.B., Koebnik R. (2009). The type III effectors of *Xanthomonas*. Mol. Plant Pathol..

[19] Ji Z., Ji C., Liu B., Zou L., Chen G., Yang B. (2016). Interfering TAL effectors of *Xanthomonas oryzae* neutralize R-gene-mediated plant disease resistance. Nat. Commun..

[20] Lee M.Y., Kim H.Y., Lee S., Kim J.G., Suh J.W., Lee C.H. (2015). Metabolomics-Based Chemotaxonomic Classification of Streptomyces spp. and Its Correlation with Antibacterial Activity. J. Microbiol. Biotechnol..

[21] Salzberg S.L., Sommer D.D., Schatz M.C., Phillippy A.M., Rabinowicz P.D., Tsuge S., Furutani A., Ochiai H., Delcher A.L., Kelley D. (2008). Erratum to: Genome sequence and rapid evolution of the rice pathogen *Xanthomonas oryzae* pv. oryzae PXO99A. BMC Genom..

[22] Ochiai H., Inoue Y., Takeya M., Sasaki A., Kaku H. (2005). Genome Sequence of *Xanthomonas oryzae* pv. oryzae Suggests Contribution of Large Numbers of Effector Genes and Insertion Sequences to Its Race Diversity. Jarq-Jpn. Agric. Res. Q..

[23] White F.F., Yang B. (2009). Host and pathogen factors controlling the rice-*Xanthomonas oryzae* interaction. Plant Physiol..

[24] Song C., Yang B. (2010). Mutagenesis of 18 type III effectors reveals virulence function of XopZ(PXO99) in *Xanthomonas oryzae* pv. oryzae. Mol. Plant Microbe Interact..

[25] Kearney B., Staskawicz B.J. (1990). Widespread distribution and fitness contribution of *Xanthomonas campestris* avirulence gene avrBs2. Nature.

[26] Swords K.M., Dahlbeck D., Kearney B., Roy M., Staskawicz B.J. (1996). Spontaneous and induced mutations in a single open reading frame alter both virulence and avirulence in *Xanthomonas campestris* pv. vesicatoria avrBs2. J. Bacteriol..

[27] Long J., Song C., Yan F., Zhou J., Zhou H., Yang B. (2018). Non-TAL Effectors From *Xanthomonas oryzae* pv. oryzae Suppress Peptidoglycan-Triggered MAPK Activation in Rice. Front. Plant Sci..

[28] Dodds P.N., Rathjen J.P. (2010). Plant immunity: Towards an integrated view of plant-pathogen interactions. Nat. Rev. Genet..

[29] Zhao Q., Bao J., Li H., Hu W., Kong Y., Zhong Y., Fu Q., Xu G., Liu F., Jiao X. (2024). Structural and biochemical basis of FLS2-mediated signal activation and transduction in rice. Plant Commun..

[30] Yoshimura S., Yamanouchi U., Katayose Y., Toki S., Wang Z.X., Kono I., Kurata N., Yano M., Iwata N., Sasaki T. (1998). Expression of Xa1, a bacterial blight-resistance gene in rice, is induced by bacterial inoculation. Proc. Natl. Acad. Sci. USA.

[31] Kumar A., Kumar R., Sengupta D., Das S.N., Pandey M.K., Bohra A., Sharma N.K., Sinha P., Sk H., Ghazi I.A. (2020). Deployment of Genetic and Genomic Tools Toward Gaining a Better Understanding of Rice-*Xanthomonas oryzae* pv. oryzae Interactions for Development of Durable Bacterial Blight Resistant Rice. Front. Plant Sci..

[32] Ji C., Ji Z., Liu B., Cheng H., Liu H., Liu S., Yang B., Chen G. (2020). Xa1 Allelic R Genes Activate Rice Blight Resistance Suppressed by Interfering TAL Effectors. Plant Commun..

[33] Zhang B., Han X., Yuan W., Zhang H. (2022). TALEs as double-edged swords in plant-pathogen interactions: Progress, challenges, and perspectives. Plant Commun..

[34] Gu K., Yang B., Tian D., Wu L., Wang D., Sreekala C., Yang F., Chu Z., Wang G.L., White F.F. (2005). R gene expression induced by a type-III effector triggers disease resistance in rice. Nature.

[35] Tian D., Wang J., Zeng X., Gu K., Qiu C., Yang X., Zhou Z., Goh M., Luo Y., Murata-Hori M. (2014). The rice TAL effector-dependent resistance protein XA10 triggers cell death and calcium depletion in the endoplasmic reticulum. Plant Cell.

[36] Wang C., Zhang X., Fan Y., Gao Y., Zhu Q., Zheng C., Qin T., Li Y., Che J., Zhang M. (2014). XA23 is an executor R protein and confers broad-spectrum disease resistance in rice. Mol. Plant.

[37] Chen X., Liu P., Mei L., He X., Chen L., Liu H., Shen S., Ji Z., Zheng X., Zhang Y. (2021). Xa7, a new executor R gene that confers durable and broad-spectrum resistance to bacterial blight disease in rice. Plant Commun..

[38] Cai L., Cao Y., Xu Z., Ma W., Zakria M., Zou L., Cheng Z., Chen G. (2017). A Transcription Activator-Like Effector Tal7 of *Xanthomonas oryzae* pv. oryzicola Activates Rice Gene Os09g29100 to Suppress Rice Immunity. Sci. Rep..

[39] Moscou M.J., Bogdanove A.J. (2009). A simple cipher governs DNA recognition by TAL effectors. Science.

[40] Zhang B., Zhang H., Li F., Ouyang Y., Yuan M., Li X., Xiao J., Wang S. (2020). Multiple Alleles Encoding Atypical NLRs with Unique Central Tandem Repeats in Rice Confer Resistance to *Xanthomonas oryzae* pv. oryzae. Plant Commun..

[41] Luo D., Huguet-Tapia J.C., Raborn R.T., White F.F., Brendel V.P., Yang B. (2021). The Xa7 resistance gene guards the rice susceptibility gene SWEET14 against exploitation by the bacterial blight pathogen. Plant Commun..

[42] Wang C., Zhang X., Fan Y., Gao Y., Zhu Q., Zheng C., Qin T., Li Y., Che J., Zhang M. (2015). XA23 is an executor R protein and confers broad-spectrum disease resistance in rice. Mol. Plant.

[43] Chu Z., Fu B., Yang H., Xu C., Li Z., Sanchez A., Park Y.J., Bennetzen J.L., Zhang Q., Wang S. (2006). Targeting xa13, a recessive gene for bacterial blight resistance in rice. Theor. Appl. Genet..

[44] Zhou J., Peng Z., Long J., Sosso D., Liu B., Eom J.S., Huang S., Liu S., Vera Cruz C., Frommer W.B. (2015). Gene targeting by the TAL effector PthXo2 reveals cryptic resistance gene for bacterial blight of rice. Plant J..

[45] Hutin M., Sabot F., Ghesquière A., Koebnik R., Szurek B. (2015). A knowledge-based molecular screen uncovers a broad-spectrum *OsSWEET14* resistance allele to bacterial blight from wild rice. Plant J..

[46] Sugio A., Yang B., Zhu T., White F.F. (2007). Two type III effector genes of *Xanthomonas oryzae* pv. oryzae control the induction of the host genes OsTFIIAgamma1 and OsTFX1 during bacterial blight of rice. Proc. Natl. Acad. Sci. USA.

[47] Zou H., Zhao W., Zhang X., Han Y., Zou L., Chen G. (2010). Identification of an avirulence gene, avrxa5, from the rice pathogen *Xanthomonas oryzae* pv. oryzae. Sci. China Life Sci..

[48] Jiang G.H., Xia Z.H., Zhou Y.L., Wan J., Li D.Y., Chen R.S., Zhai W.X., Zhu L.H. (2006). Testifying the rice bacterial blight resistance gene xa5 by genetic complementation and further analyzing xa5 (Xa5) in comparison with its homolog TFIIAgamma1. Mol. Genet. Genom..

[49] Yang B., Sugio A., White F.F. (2006). Os8N3 is a host disease-susceptibility gene for bacterial blight of rice. Proc. Natl. Acad. Sci. U S A.

[50] Antony G., Zhou J., Huang S., Li T., Liu B., White F., Yang B. (2010). Rice xa13 recessive resistance to bacterial blight is defeated by induction of the disease susceptibility gene Os-11N3. Plant Cell.

[51] Yu Y., Streubel J., Balzergue S., Champion A., Boch J., Koebnik R., Feng J., Verdier V., Szurek B. (2011). Colonization of rice leaf blades by an African strain of *Xanthomonas oryzae* pv. oryzae depends on a new TAL effector that induces the rice nodulin-3 Os11N3 gene. Mol. Plant Microbe Interact..

[52] Streubel J., Pesce C., Hutin M., Koebnik R., Boch J., Szurek B. (2013). Five phylogenetically close rice SWEET genes confer TAL effector-mediated susceptibility to *Xanthomonas oryzae* pv. oryzae. New Phytol..

[53] Xu Z., Xu X., Gong Q., Li Z., Li Y., Wang S., Yang Y., Ma W., Liu L., Zhu B. (2019). Engineering Broad-Spectrum Bacterial Blight Resistance by Simultaneously Disrupting Variable TALE-Binding Elements of Multiple Susceptibility Genes in Rice. Mol. Plant.

[54] Yuan M., Ke Y., Huang R., Ma L., Yang Z., Chu Z., Xiao J., Li X., Wang S. (2016). A host basal transcription factor is a key component for infection of rice by TALE-carrying bacteria. Elife.

[55] Tran T.T., Pérez-Quintero A.L., Wonni I., Carpenter S.C.D., Yu Y., Wang L., Leach J.E., Verdier V., Cunnac S., Bogdanove A.J. (2018). Functional analysis of African *Xanthomonas oryzae* pv. oryzae TALomes reveals a new susceptibility gene in bacterial leaf blight of rice. PLoS Pathog..

[56] Cernadas R.A., Doyle E.L., Niño-Liu D.O., Wilkins K.E., Bancroft T., Wang L., Schmidt C.L., Caldo R., Yang B., White F.F. (2014). Code-assisted discovery of TAL effector targets in bacterial leaf streak of rice reveals contrast with bacterial blight and a novel susceptibility gene. PLoS Pathog..

[57] Zhao B., Lin X., Poland J., Trick H., Leach J., Hulbert S. (2005). A maize resistance gene functions against bacterial streak disease in rice. Proc. Natl. Acad. Sci. U S A.

[58] Zhou Y.L., Xu M.R., Zhao M.F., Xie X.W., Zhu L.H., Fu B.Y., Li Z.K. (2010). Genome-wide gene responses in a transgenic rice line carrying the maize resistance gene Rxo1 to the rice bacterial streak pathogen, X*anthomonas oryzae* pv. oryzicola. BMC Genom..

[59] Read A.C., Rinaldi F.C., Hutin M., He Y.Q., Triplett L.R., Bogdanove A.J. (2016). Suppression of Xo1-Mediated Disease Resistance in Rice by a Truncated, Non-DNA-Binding TAL Effector of *Xanthomonas oryzae*. Front. Plant Sci..

[60] Triplett L.R., Cohen S.P., Heffelfinger C., Schmidt C.L., Huerta A.I., Tekete C., Verdier V., Bogdanove A.J., Leach J.E. (2016). A resistance locus in the American heirloom rice variety Carolina Gold Select is triggered by TAL effectors with diverse predicted targets and is effective against African strains of *Xanthomonas oryzae* pv. oryzicola. Plant J..

[61] Read A.C., Hutin M., Moscou M.J., Rinaldi F.C., Bogdanove A.J. (2020). Cloning of the Rice Xo1 Resistance Gene and Interaction of the Xo1 Protein with the Defense-Suppressing *Xanthomonas* Effector Tal2h. Mol. Plant Microbe Interact..

[62] Chen L.Q., Hou B.H., Lalonde S., Takanaga H., Hartung M.L., Qu X.Q., Guo W.J., Kim J.G., Underwood W., Chaudhuri B. (2010). Sugar transporters for intercellular exchange and nutrition of pathogens. Nature.

[63] Yuan T., Li X., Xiao J., Wang S. (2011). Characterization of *Xanthomonas oryzae*-responsive cis-acting element in the promoter of rice race-specific susceptibility gene Xa13. Mol. Plant.

[64] Blanvillain-Baufumé S., Reschke M., Solé M., Auguy F., Doucoure H., Szurek B., Meynard D., Portefaix M., Cunnac S., Guiderdoni E. (2017). Targeted promoter editing for rice resistance to *Xanthomonas oryzae* pv. oryzae reveals differential activities for SWEET14-inducing TAL effectors. Plant Biotechnol. J..

[65] Liu Q., Yuan M., Zhou Y., Li X., Xiao J., Wang S. (2011). A paralog of the MtN3/saliva family recessively confers race-specific resistance to *Xanthomonas oryzae* in rice. Plant Cell Env..

[66] Chu Z., Yuan M., Yao J., Ge X., Yuan B., Xu C., Li X., Fu B., Li Z., Bennetzen J.L. (2006). Promoter mutations of an essential gene for pollen development result in disease resistance in rice. Genes. Dev..

[67] Ma W., Zou L., Zhiyuan J.I., Xiameng X.U., Zhengyin X.U., Yang Y., Alfano J.R., Chen G. (2018). *Xanthomonas oryzae* pv. oryzae TALE proteins recruit OsTFIIAγ1 to compensate for the absence of OsTFIIAγ5 in bacterial blight in rice. Mol. Plant Pathol..

[68] Xu X., Xu Z., Ma W., Haq F., Li Y., Shah S.M.A., Zhu B., Zhu C., Zou L., Chen G. (2021). TALE-triggered and iTALE-suppressed Xa1-mediated resistance to bacterial blight is independent of rice transcription factor subunits OsTFIIAγ1 or OsTFIIAγ5. J. Exp. Bot..

[69] Xie X., Chen Z., Cao J., Guan H., Lin D., Li C., Lan T., Duan Y., Mao D., Wu W. (2014). Toward the positional cloning of qBlsr5a, a QTL underlying resistance to bacterial leaf streak, using overlapping sub-CSSLs in rice. PLoS One.

[70] Cheong H., Kim C.Y., Jeon J.S., Lee B.M., Sun Moon J., Hwang I. (2013). *Xanthomonas oryzae* pv. oryzae type III effector XopN targets OsVOZ2 and a putative thiamine synthase as a virulence factor in rice. PLoS One.

[71] Liao Z.-X., Li J.-Y., Mo X.-Y., Ni Z., Jiang W., He Y.-Q., Huang S. (2020). Type III effectors xopN and avrBS2 contribute to the virulence of *Xanthomonas oryzae* pv. oryzicola strain GX01. Res. Microbiol..

[72] Wang S., Sun J., Fan F., Tan Z., Zou Y., Lu D. (2016). A *Xanthomonas oryzae* pv. oryzae effector, XopR, associates with receptor-like cytoplasmic kinases and suppresses PAMP-triggered stomatal closure. Sci. China Life Sci..

[73] Yamaguchi K., Yamada K., Ishikawa K., Yoshimura S., Hayashi N., Uchihashi K., Ishihama N., Kishi-Kaboshi M., Takahashi A., Tsuge S. (2013). A receptor-like cytoplasmic kinase targeted by a plant pathogen effector is directly phosphorylated by the chitin receptor and mediates rice immunity. Cell Host Microbe.

[74] Yamaguchi K., Nakamura Y., Ishikawa K., Yoshimura Y., Tsuge S., Kawasaki T. (2013). Suppression of rice immunity by *Xanthomonas oryzae* type III effector Xoo2875. Biosci. Biotechnol. Biochem..

[75] Qin J., Zhou X., Sun L., Wang K., Yang F., Liao H., Rong W., Yin J., Chen H., Chen X. (2018). The *Xanthomonas* effector XopK harbours E3 ubiquitin-ligase activity that is required for virulence. New Phytol..

[76] Ishikawa K., Yamaguchi K., Sakamoto K., Yoshimura S., Inoue K., Tsuge S., Kojima C., Kawasaki T. (2014). Bacterial effector modulation of host E3 ligase activity suppresses PAMP-triggered immunity in rice. Nat. Commun..

[77] Ma W., Xu X., Cai L., Cao Y., Haq F., Alfano J.R., Zhu B., Zou L., Chen G. (2020). A *Xanthomonas oryzae* type III effector XopL causes cell death through mediating ferredoxin degradation in Nicotiana benthamiana. Phytopathol. Res..

[78] Ji H., Li T., Li X., Li J., Yu J., Zhang X., Liu D. (2022). XopZ and ORP1C cooperate to regulate the virulence of *Xanthomonas oryzae* pv. oryzae on Nipponbare. Plant Signal Behav..

[79] Fiehn O., Kopka J., Dörmann P., Altmann T., Trethewey R.N., Willmitzer L. (2000). Metabolite profiling for plant functional genomics. Nat. Biotechnol..

[80] Finkelstein D., Ewing R., Gollub J., Sterky F., Cherry J.M., Somerville S. (2002). Microarray data quality analysis: Lessons from the AFGC project. Arabidopsis Functional Genomics Consortium. Plant Mol. Biol..

[81] Henikoff S., Comai L. (2003). Single-nucleotide mutations for plant functional genomics. Annu. Rev. Plant Biol..

[82] Sana T.R., Fischer S., Wohlgemuth G., Katrekar A., Jung K.H., Ronald P.C., Fiehn O. (2010). Metabolomic and transcriptomic analysis of the rice response to the bacterial blight pathogen *Xanthomonas oryzae* pv. oryzae. Metabolomics.

[83] Jiang N., Fu J., Zeng Q., Liang Y., Shi Y., Li Z., Xiao Y., He Z., Wu Y., Long Y. (2021). Genome-wide association mapping for resistance to bacterial blight and bacterial leaf streak in rice. Planta.

[84] Lu J., Wang C., Zeng D., Li J., Shi X., Shi Y., Zhou Y. (2021). Genome-Wide Association Study Dissects Resistance Loci against Bacterial Blight in a Diverse Rice Panel from the 3000 Rice Genomes Project. Rice.

[85] Zhang F., Wu Z.C., Wang M.M., Zhang F., Dingkuhn M., Xu J.L., Zhou Y.L., Li Z.K. (2017). Genome-wide association analysis identifies resistance loci for bacterial blight in a diverse collection of indica rice germplasm. PLoS One.

[86] Wu G., Zhang Y., Wang B., Li K., Lou Y., Zhao Y., Liu F. (2021). Proteomic and Transcriptomic Analyses Provide Novel Insights into the Crucial Roles of Host-Induced Carbohydrate Metabolism Enzymes in *Xanthomonas oryzae* pv. oryzae Virulence and Rice-Xoo Interaction. Rice.

[87] Zhao Y., Qian G., Yin F., Fan J., Zhai Z., Liu C., Hu B., Liu F. (2011). Proteomic analysis of the regulatory function of DSF-dependent quorum sensing in *Xanthomonas oryzae* pv. oryzicola. Microb. Pathog..

[88] Zhang F., Zhang F., Huang L., Zeng D., Cruz C.V., Li Z., Zhou Y. (2020). Comparative proteomic analysis reveals novel insights into the interaction between rice and *Xanthomonas oryzae* pv. oryzae. BMC Plant Biol..

[89] Bi Y., Yu Y., Mao S., Wu T., Wang T., Zhou Y., Xie K., Zhang H., Liu L., Chu Z. (2024). Comparative transcriptomic profiling of the two-stage response of rice to *Xanthomonas oryzae* pv. oryzicola interaction with two different pathogenic strains. BMC Plant Biol..

[90] Sahu A., Das A., Saikia K., Barah P. (2020). Temperature differentially modulates the transcriptome response in Oryza sativa to *Xanthomonas oryzae* pv. oryzae infection. Genomics.

[91] Das P.P., Kumar A., Mohammed M., Bhati K., Babu K.R., Bhandari K.P., Sundaram R.M., Ghazi I.A. (2025). Comparative metabolites analysis of resistant, susceptible and wild rice species in response to bacterial blight disease. BMC Plant Biol..

[92] Chen P., Wang J., Liu Q., Liu J., Mo Q., Sun B., Mao X., Jiang L., Zhang J., Lv S. (2024). Transcriptome and Metabolome Analysis of Rice Cultivar CBB23 after Inoculation by *Xanthomonas oryzae* pv. oryzae Strains AH28 and PXO99(A). Plants.

[93] Vo K.T.X., Rahman M.M., Rahman M.M., Trinh K.T.T., Kim S.T., Jeon J.S. (2021). Proteomics and Metabolomics Studies on the Biotic Stress Responses of Rice: An Update. Rice.

[94] Erb M., Kliebenstein D.J. (2020). Plant Secondary Metabolites as Defenses, Regulators, and Primary Metabolites: The Blurred Functional Trichotomy. Plant Physiol..

[95] Erb M. (2018). Volatiles as inducers and suppressors of plant defense and immunity-origins, specificity, perception and signaling. Curr. Opin. Plant Biol..

[96] Bouwmeester H., Schuurink R.C., Bleeker P.M., Schiestl F. (2019). The role of volatiles in plant communication. Plant J..

[97] Yang W., Zhang L., Yang Y., Xiang H., Yang P. (2024). Plant secondary metabolites-mediated plant defense against bacteria and fungi pathogens. Plant Physiol. Biochem..

[98] Yang Y., Zhou Y., Sun J., Liang W., Chen X., Wang X., Zhou J., Yu C., Wang J., Wu S. (2022). Research Progress on Cloning and Function of Xa Genes Against Rice Bacterial Blight. Front. Plant Sci..

[99] Hou Y., Liang Y., Yang C., Ji Z., Zeng Y., Li G., E Z. (2023). Complete Genomic Sequence of *Xanthomonas oryzae* pv. oryzae Strain, LA20, for Studying Resurgence of Rice Bacterial Blight in the Yangtze River Region, China. Int. J. Mol. Sci..

[100] Gupta A., Liu B., Chen Q.J., Yang B. (2023). High-efficiency prime editing enables new strategies for broad-spectrum resistance to bacterial blight of rice. Plant Biotechnol. J..

[101] Gupta A., Liu B., Raza S., Chen Q.-J., Yang B. (2024). Modularly assembled multiplex prime editors for simultaneous editing of agronomically important genes in rice. Plant Commun..

[102] Ni Z., Cao Y., Jin X., Fu Z., Li J., Mo X., He Y., Tang J., Huang S. (2021). Engineering Resistance to Bacterial Blight and Bacterial Leaf Streak in Rice. Rice.

[103] Duy P.N., Lan D.T., Pham Thu H., Thi Thu H.P., Nguyen Thanh H., Pham N.P., Auguy F., Bui Thi Thu H., Manh T.B., Cunnac S. (2021). Improved bacterial leaf blight disease resistance in the major elite Vietnamese rice cultivar TBR225 via editing of the OsSWEET14 promoter. PLoS One.

[104] Römer P., Jordan T., Lahaye T. (2010). Identification and application of a DNA-based marker that is diagnostic for the pepper (Capsicum annuum) bacterial spot resistance gene Bs3. Plant Breed..

[105] Huang F., He N., Yu M., Li D., Yang D. (2023). Identification and fine mapping of a new bacterial blight resistance gene, Xa43(t), in Zhangpu wild rice (*Oryza rufipogon*). Plant Biol..

[106] Hummel A.W., Doyle E.L., Bogdanove A.J. (2012). Addition of transcription activator-like effector binding sites to a pathogen strain-specific rice bacterial blight resistance gene makes it effective against additional strains and against bacterial leaf streak. New Phytol..

[107] Zeng X., Tian D., Gu K., Zhou Z., Yang X., Luo Y., White F.F., Yin Z. (2015). Genetic engineering of the Xa10 promoter for broad-spectrum and durable resistance to *Xanthomonas oryzae* pv. oryzae. Plant Biotechnol. J..

[108] Li Z.G., He F., Zhang Z., Peng Y.L. (2012). Prediction of protein-protein interactions between Ralstonia solanacearum and Arabidopsis thaliana. Amino Acids.

[109] Zheng C., Liu Y., Sun F., Zhao L., Zhang L. (2021). Predicting Protein-Protein Interactions Between Rice and Blast Fungus Using Structure-Based Approaches. Front. Plant Sci..

[110] Mukhtar M.S., Carvunis A.R., Dreze M., Epple P., Steinbrenner J., Moore J., Tasan M., Galli M., Hao T., Nishimura M.T. (2011). Independently evolved virulence effectors converge onto hubs in a plant immune system network. Science.

[111] Weßling R., Epple P., Altmann S., He Y., Yang L., Henz S.R., McDonald N., Wiley K., Bader K.C., Gläßer C. (2014). Convergent targeting of a common host protein-network by pathogen effectors from three kingdoms of life. Cell Host Microbe.

[112] Cao F.Y., Khan M., Taniguchi M., Mirmiran A., Moeder W., Lumba S., Yoshioka K., Desveaux D. (2019). A host-pathogen interactome uncovers phytopathogenic strategies to manipulate plant ABA responses. Plant J..

