# Correction: Qi et al. Recent Progress in Rice–*Xanthomonas oryzae* Interactions. *Biology* 2025, *14*, 471

**DOI:** 10.3390/biology14070837

**Published:** 2025-07-09

**Authors:** Yuting Qi, Qiong Rao, Chenglong Lu, Junyi Gong, Yuxuan Hou

**Affiliations:** 1 Zhejiang Key Laboratory of Biology and Ecological Regulation of Crop Pathogens and Insects, College of Advanced Agricultural Sciences, Zhejiang A&F University, Hangzhou 311300, China; 13140590443@163.com (Y.Q.); qiong.rao@zafu.edu.cn (Q.R.); 2 State Key Laboratory of Rice Biology and Breeding, China National Rice Research Institute, Hangzhou 311400, China; lclzbzkerban@163.com

- **Errors in Table 1**

In the original publication [1], there were two mistakes in Table 1 as published. The original reference [45] has been replaced by a new reference: “Hutin, M.; Sabot, F.; Ghesquière, A.; Koebnik, R.; Szurek, B. A knowledge-based molecular screen uncovers a broad-spectrum *OsSWEET14* resistance allele to bacterial blight from wild rice. *Plant J.* **2015**, *84*, 694–703”.

The original citations for references [56,57] were incorrect due to improper citation. The numbers have been updated so they correctly refer to the reference list. With this correction, the order of some references has also been adjusted accordingly. The corrected Table 1 appears below. The authors state that the scientific conclusions are unaffected. This correction was approved by the Academic Editor. The original publication has also been updated.

**Table 1:** Rice genes targeted by TALEs.

Tale-Targeted (*R*/*S* Gene)		Encoding Products	Matched TALEs	References
Resistance	*Xa1* *Xo1* *Xa2/31* *Xa14* *Xa45*	NLR	Multiple TALEs, iTALEs/truncTALE	[19,30,33,40]
	*Xa7*	Executor	AvrXa7, PthXo3	[37,41]
	*Xa10*	Executor	AvrXa10	[35]
	*Xa23*	Executor	AvrXa23	[42]
	*Xa27*	executor	AvrXa27	[34]
	*xa13*	Sweet transporter	PthXo1	[43]
	*xa25*	Sweet transporter	PthXo2	[44]
	*xa41*	Sweet transporter	AvrXa7, PthXo3, Tal5, TalC	[45]
	*xa5*	TFIIA transcription factor	AvrXa5, PthXo7	[46,47,48]
Susceptibility	*OsSWEET11(Xa13/Os8N3)*	Sweet transporter	PthXo1	[49]
	*OsSWEET14(Xa41/Os11N3)*	Sweet transporter	AvrXa7, PthXo3, TalC, Tal5	[50,51,52]
	*OsSWEET13(Xa25/Os12N3)*	Sweet transporter	PthXo2	[44,53]
	*OsSWEET12*	Sweet transporter	ArtTAL12	[52]
	*OsSWEET15*	Sweet transporter	ArtTAL15	[52]
	*OsTFIIAγ5*	Gamma subunit of rice basal transcription factor	Multiple TALEs	[54]
	*OsTFIIAγ* *1*	Gamma subunit of rice basal transcription factor	PthXo7	[46]
	*OsTFX1*	bZIP transcription factor	PthXo6 TalB_MAl1_	[46,55]
	*OsERF#123*	AP2/ERF transcription factor	TalB_MAl1_	[55]
	*OsSULTR3;6*	Sulfate transporter	Tal2g	[56]

- **Errors in Table 2**

In the original publication, there were two mistakes in Table 2 as published. The citation for reference [74] on the line of *OsRLCK185* was wrong. This citation has been changed to reference [73]: “Yamaguchi, K.; Yamada, K.; Ishikawa, K.; Yoshimura, S.; Hayashi, N.; Uchihashi, K.; Ishihama, N.; Kishi-Kaboshi, M.; Takahashi, A.; Tsuge, S.; et al. A receptor-like cytoplasmic kinase targeted by a plant pathogen effector is directly phosphorylated by the chitin receptor and mediates rice immunity. *Cell Host Microbe* **2013**, *13*, 347–357.” Additionally, a typo was present, and “*OsSERK1*” has been changed to “*OsSERK2*”. The corrected Table 2 appears below.

**Table 2:** Rice genes interacted with non-TALEs.

Rice Genes (Interaction Genes)	Encoding Products	Matched TALEs	References
* OsVOZ2, OsXNP *	Vascular plant one zinc finger protein 2, putative thiamine synthase	XopN	[27,70,71]
*OsBIK1*	Receptor-like kinases	XopR	[72]
* OsRLCK185 *	Receptor-like kinase	XopY	[73]
* OsBAK1 *	Receptor-like kinase	XopAA	[74]
* OsSERK2 *	Somatic embryogenic receptor kinase 2	XopK	[75]
* OsPUB44 *	Ubiquitin E3 ligase	XopP	[76]
* NbFd *	Ferredoxin protein	XopL	[77]
* OsORP1C *	Oxysterol-binding related protein	XopZ	[78]

- **Text Correction**

There was an error in the original publication. The citation for reference [100] in the following paragraph was not relevant: “The disruption of the binding elements for PthXo3, AvrXa7, and PthXo2 within the promoter regions of the *OsSWEET14* and *OsSWEET13* genes through TALEN technology has demonstrated significant resistance to BB [44,100].”

A correction has been made to Section 8, Paragraph 3, as follows:

“The disruption of the binding elements for PthXo3, AvrXa7, and PthXo2 within the promoter regions of the *OsSWEET14* and *OsSWEET13* genes through TALEN technology has demonstrated significant resistance to BB [44].”

- **Wrong Reference**

Reference 45 has been replaced with the following new reference: “Hutin, M.; Sabot, F.; Ghesquière, A.; Koebnik, R.; Szurek, B. A knowledge-based molecular screen uncovers a broad-spectrum *OsSWEET14* resistance allele to bacterial blight from wild rice. *Plant J.* **2015**, *84*, 694–703”.

## References

[1] Qi Y., Rao Q., Lu C., Gong J., Hou Y. (2025). Recent Progress in Rice–*Xanthomonas oryzae* Interactions. Biology.

